# Fatty Acid Indices and the Nutritional Properties of *Karakul* Sheep Meat

**DOI:** 10.3390/nu15041061

**Published:** 2023-02-20

**Authors:** Otilia Cristina Murariu, Florin Murariu, Gabriela Frunză, Marius Mihai Ciobanu, Paul Corneliu Boișteanu

**Affiliations:** 1 Department of Food Technology, Faculty of Agriculture, “Ion Ionescu de la Brad” University of Life Sciences, 700490 Iasi, Romania; 2 Department of Agroeconomy, Faculty of Agriculture, “Ion Ionescu de la Brad” University of Life Sciences, 700490 Iasi, Romania; 3 Department of Control, Expertise and Services, Faculty of Food and Animal Sciences, “Ion Ionescu de la Brad” University of Life Sciences, 700490 Iasi, Romania

**Keywords:** food quality, sheep meat, nutritional properties, human health

## Abstract

This study aimed to evaluate the fatty acid profile and health lipid indices of sheep meat (from 52 *Karakul* sheep from NE Romania). The effect of age at slaughter and the influence of muscle region were studied for nutritional parameters, especially the fatty acids from lipid fractions. Based on the fatty acid profiles and lipid contents, the sanogenic indices were determined for two sheep muscle groups. Thus, two different muscle regions from lamb and adult sheep were analysed from both genders, the *Longissimus dorsi* and *Triceps brachii*, to argue the advantages of each category and the rationalization, in terms of meat consumption, regarding their impact on human health. Sheep meat has many components with beneficial effects on human health. Apart from the fact that it is an important source of nutrients due to its high content of proteins, lipids, and minerals, it is also a product that can provide fundamental bioactive compounds for maintaining metabolic functions. The qualitative indices assessment revealed that lambs have meat with high PUFA content on *Longissimus dorsi* muscles (approx. 25% of total fatty acids), 0.68 for PUFA/SFA, with highest values for n-3 (approx. 8%) and n-6 (approx. 14%). Appropriate values can also be observed in *Triceps brachii* muscles from adult sheep. The sanogenic indices also presented good values for *Longissimus dorsi* from lambs and *Triceps brachii* from adult sheep (polyunsaturation index = 7.2–10.2; atherogenic index = 0.56–0.67; thrombogenic index = 0.78–0.96; hypocholesterolemic/hypercholesterolemic index = 2.4–2.7 (for *Longissimus dorsi*)).

## 1. Introduction

Over the past century, considerable progress has been made in improving human welfare worldwide [1]. However, the shifting of livestock production from the past century that aimed to ensure large quantities of high-value proteins and lipids to feed the population [2,3] is not sufficient for the future when considering the accelerated growth of the world population and increased life expectancy that could lead to a future “food crisis” [4]. Additionally, the impact of climate change is already being felt, highlighting the need for solutions for the sustainability of food and agricultural systems. The global population is predicted to reach more than 9.7 billion by mid-century [5], suggesting a necessity to respond with enough food to eradicate hunger by 2030 [1].

The domestic sheep (*Ovis aries*) is a very widespread ruminant species, a *Pleistocene* mammal, raised primarily for its meat, milk, wool, and hides [1]. Great interest has been shown in the lipid composition studies of sheep meat, being recognized as an important source of n-3 and n-6 fatty acids [6].

Polyunsaturated fatty acids (PUFA) that belong to either of the two distinct and non-interconvertible series n-6 and n-3 are essential nutrients for humans [7]. The metabolic precursors of these two series, linoleic and α-linolenic acids are essential nutrients [8]. Once absorbed, these two fatty acids lead to the synthesis of, and specific incorporation into virtually all cell membranes of active long-chain PUFA derivatives, such as arachidonic acid for the n-6 series and eicosapentaenoic and docosahexaenoic acids for the n-3 series [9]. Long-chain PUFA affect many physiological functions, because they are essential factors in many cellular activities, by regulating the physical properties of membranes, eicosanoid signalling, and the encoding gene expression of triglyceride storage enzymes and fatty acid oxidation [7]. Consumption of n-6 and n-3 fatty acids in balanced proportions could be crucial in regulating cellular physiology and preventing pathologies such as cardiovascular, autoimmune, and inflammatory diseases, diabetes and obesity, and certain neuropsychiatric diseases [10]. Dietary intake of n-3 fatty acids (linoleic and docosahexaenoic) is crucial for retinal development and neural function in newborns [7]. The daily consumption of fatty acids in an adult is approximately 95 g, which represents more than one-third of the metabolizable caloric energy present in foods such as vegetable oils, butter, margarine, meat, sausage, fish, and eggs [11]. After their intestinal absorption, which under normal physiological conditions represents more than 90% of the ingested percentage, fatty acids are incorporated into the bloodstream in the form of lipoproteins that distribute them to the user tissue (adipose tissue, muscle, and liver) [12]. They are metabolized in the liver and then redistributed in the body in the form of denser lipoproteins [13]. All fatty acids, whether they come from food or liver metabolism, have a structural function as a fundamental constituent of cell membranes and an energetic function related to their enzymatic catabolism (β-oxidation), which takes place mainly in the mitochondria. The nature of fatty acids can modulate their distribution to one or other of these metabolic pathways [7]. Some of them play a specific role in essential nutrition. These are the polyunsaturated fatty acids of the n-6 and n-3 series, whose two essential metabolic precursors are linoleic acid (18:2n-6) and ɑ-linolenic acid (18:3n-3) [9].

Meat, derived from various species of poultry, mammals, and fish, counts among the top preferences of consumers and producers [3,6,14,15]. This situation is present due to the animals’ ability to efficiently capitalise on various roughage raw materials, or even secondary products and residues of agricultural nature, which are unfit for human consumption, transforming them into a product of high nutritional–biological value: meat [16]. Meat represents a complete foodstuff, like eggs and milk, possessing an important plastic and energetic role [17]. These assessments are necessary, considering modern consumers who are increasingly concerned with meat production under safe conditions with no adverse effects on their health. This leads to a high preference for meat produced under natural conditions or ecological products. Ecological meat production implies minimal use of chemical substances in sheep breeding activities [18].

Since food quality, implicitly the level and quality of nutrients from food, is a primordial factor that counts in the assessment of the state of health, the raw material-producing industry must take into consideration all possible ways of improving the nutritional value of foods. However, data on some qualitative indexes of sheep meat, which could otherwise contribute to a larger picture of the nutritional–biological properties of this product, occurs infrequently in the literature, especially when consumers are interested in the meat presently commercialized and consumed in Romania.

Sheep meat consists of a mixture of several types of tissues: muscular, adipose, bone, loose, fibrous, cartilaginous, nervous, and epithelial. The percentage of these tissues in the composition of the meat is determined by genotype, species, breed, age, gender, physiological state, fattening state, diet, or by muscle region. The chemical properties of meat are given by the properties of its component elements, giving it a solid nutritional potential through its high content of proteins and lipids and low levels of carbohydrates, including glucose and glycogen [19,20]. Sheep meat is considered a functional food due to its high nutritional properties with a high essential amino acids content [17] and its sanogenic lipid profile. Meat lipids consist of triglycerides (forming intramuscular fat) and phospholipids, which provide the two essential precursors and long-chain derivatives, particularly those of the n-3 series. The lipid content of meat and its fatty acid composition varies according to the anatomical location of the muscle and the animal species’ diet. 18:2n-6 is the main PUFA present in the fat part of the meat (from 150 mg/100 g for veal to about 2800 mg/100 g for pork), with 18:3n-3 generally being in the minority, except in horse and rabbit meat. Meat also provides substantial amounts of long-chain n-3 PUFA (40 to 120 mg/100 g) [21]. The amount of fat in the human diet and especially the amount of saturated fatty acids have been considered a major risk factor for coronary heart disease [22]. Some PUFA (n-6, n-3, and conjugated linoleic acid) present in animal muscle tissue seem to play a favourable role through nutrition in preventing or reducing some human diseases. Therefore, the PUFA/SFA and n-6/n-3 fatty acids are considered important indices for the nutritional evaluation of fatty meat [4,23]. Carcass weight is an essential criterion for establishing the commercial category of lambs/sheep; an important factor that influences both meat quality [23] and consumer preferences [24].

Within the present context, our results contribute to the most detailed understanding of Karakul sheep meat (from 52 NE Romania *Karakul* sheep) regarding the implications of its fatty acid contents on human health. This study also analysed the effects of muscle region and slaughter age on nutritional parameters, with a particular focus on the fatty acids from lipid fractions.

## 2. Materials and Methods

### 2.1. Sample Collection

The animals used in this research were of the *Karakul* sheep breed, raised in a traditional system on a Horlesti farm located near Iasi city, in the NE of Romania. In the favourable seasons (spring, summer, and autumn), the animals were fed with a green meal, by grazing, and in the cold season, they were reared in stalls, being fed with hay and cereals. The sheep were exploited for milk, meat, and pelt production.

To obtain biological material, the animals were slaughtered in a slaughterhouse with a special slaughter line for swine in Bacau city approved by the EU; twelve sheep were slaughtered after feed withdrawal. The sheep were transported to the slaughterhouse in special animal transport vehicles. The transportation took around 3 h. The animals received for slaughter were left to rest for 8–12 h at the slaughterhouse in an especially organised sheep-holding pen occupying 447.3 m^2^. The unit has implemented and maintained a self-monitoring programme based on HACCP principles, being certified by the Food Safety Management System according to ISO 22000 and with a Quality Management System according to ISO 9001.

The investigated biological material was collected from sheep and grouped into the following experimental categories according to the weight at slaughter: lambs of the *Karakul* breed (n = 26, aged 10–12 weeks and carcasses weight at 11–13 kg) and adult sheep (13 male and 13 female) of the *Karakul* breed (n = 26, 20–24 months, carcasses weight (20–30 kg).

After slaughter, the carcasses were kept at a temperature of 10–14 °C for 6 h before refrigeration, then stored for 18 h at 4 °C. After 24 h of chilling, the carcasses were weighed and the samples of *Longissimus dorsi* and *Triceps brachii* were collected (three samples for each animal and each muscle). The samples were subsampled and the analyses for chemical components were performed in triplicates and for fatty acids in duplicates.

Collection, sampling, identification, and preservation of samples are very important steps with major influences on the results obtained from laboratory analysis. The *Longissimus dorsi* and *Triceps brachii* muscles were excised for analysis.

The *Longissimus dorsi* (L.d.) muscle is the major extensor muscle of the spine, occupying the entire vertebrocostal trough. To sample the *Longissimus dorsi* muscle, a section was taken between the 10th and 11th thoracic vertebra and between the 4th and 5th lumbar vertebra.

The *Triceps brachii* (T.b.) muscle is the strongest muscle of the thoracic limb, occupying the entire space between the caudal edges of the shoulder blade, humerus, and olecranon. To sample the *Triceps brachii* muscle, a section was made across the calf from the mid-humerus to the radius.

The samples were transported in a refrigerated state (4 °C) to the laboratory and immediately analyzed in duplicate to determine the chemical composition and the lipid amounts.

### 2.2. The Chemical Components and Energy Values Determinations

The major chemical contents (water, proteins, and lipids) were determined through the spectrophotometric method using the Omega Bruins Food-Check^®^ (NIR) spectrophotometer (Bruins Instruments^®^ GmbH, Puchheim, Germany). Thus, 100 g of finely ground and homogenized tissue samples were placed on the glass plate and inserted in the spectrophotometer. The reflection values were calculated from the ratio of the values read by the analyzer and the reference values, which represent the basis for the calculation of the analyzed parameters; the limits of the working spectral range were 730–1100 nm [25].

The total dry matter content was determined by drying about 5 g of fresh and ground homogenized meat samples in a forced air-drying oven (Biobase^®^, Jinan, China) at 105 °C for 12 h.

The ash content was determined by furnace muffle calcination in a Nabertherm B180^®^ device, (Germany) (550° for 24 h after preliminary carbonization in a bunsen burner flame [26]). The nitrogen-free extract was calculated using Equation (1):

NFE (%) = 100 − H_2_O(%) − Proteins(%) − Lipids(%) − Ash(%), (1)

The gross energy was determined using the Atwater Equation (2), using the percentage value of each organic matter compound (proteins, lipids, and nitrogen-free extract).

GE (kcal/100 g F.W.) = 5.7 kcal × g Proteins + 9.5 kcal × g Lipids + 4.2 Kcal × g NFE.(2)

### 2.3. Sample Preparation

To determine the fatty acids, 150 g of meat was sampled from the muscle tissue of interest, and the connective tissue was removed (so as not to remove intramuscular and infiltrated fat). A 10 g portion of the sample was vacuum-packed and frozen at −20 °C at the time of analysis. The freeze-drying of the meat samples was carried out in the following standard steps: the liquid material was frozen in a condenser of the Hetosicc CD4^®^ freeze-dryer with ice to −50 °C; then, primary lyophilization of samples took place for 5 to 6 h; finally, the desorption phase was carried out at 35 °C for 12 h. After freeze-drying, the samples were subjected to hydric re-equilibration (by keeping them at atmospheric conditions for 12 h to absorb chemically bound water) and then ground to a fine powder using a blender.

### 2.4. Determination of the Fatty Acid Profile of Lipids

The detection of fatty acids was possible after the derivatization of the lipid extract with acetyl chloride and methanol, which liberates fatty acids from triglycerides with the formation of methyl esters, detectable by FID (flame ionization detector).

#### 2.4.1. Total Lipid Extraction

Total lipids were extracted with a chloroform–methanol mixture (2:1 *v*/*v*; [27]) using the Ultraturax^®^ instrument. The transmethylation was made according to ISO 2000 methods [28] and the fatty acid methyl esters were determined using a flame ionization detector in a gas chromatography system (Carlo Erba Instrument, Carlo Erba Reagents, Milano, Italy).

#### 2.4.2. Separation and Detection of Fatty Acid Methyl Esters by Gas Chromatography

Gas chromatographic conditions: Omega Wax 320 capillary column (30 m × 0.32 mm × 0.25 µm film thickness), fragmentation ratio 1:50, flame ionizing detector temperature and injector temperature were 260 °C. Running time was 43 min with the temperature ramp programmed from 160 °C to 260 °C, with an increase of 1 °C per minute for the first 26 min, 5 °C per minute for the next 16 min, and 1 °C for the last minute. The flow rate of the carrier gas (Helium) was 1.2 mL/min. The identification of fatty acid methyl esters was performed according to their retention time using the internal standard (IS). Nonadecanoic acid (C19:0) methyl ester was added as IS.

The analyses included the quantitative determination of their total lipid content as well as their quality, described by means of the fatty acid profile, and coded as follows: SFA—saturated fatty acids: (C8:0—caprylic acid; C10:0—caprynic acid; C11:0—undecylenic acid; C12:0—lauric acid; C14:0—myristic acid; C15:0—pentanoic acid; C16:0—palmitic acid; C17:0—heptadecanoic acid; C18:0—stearic acid; C20:0—arachic acid; C21:0—henicozanoic acid; C22:0—behenic acid; C23:0—trichozanoic acid; and C24:0—lignoceric acid), MUFA—monounsaturated acids: (C14:1—myristoleic acid; C15:1-*10-cis*-pentadecanoic acid; C16:1n-7-palmitoleic acid; C17:1-10-*cis* heptadecanoic acid; C18:1n-9t—*trans* oleic acid; C18:1n-9c—*cis* oleic acid; C18:1n-7—*cis* vaccenic acid; C20:1n-9—gadoleic acid; C22:1n-9—ketooleic acid; and C24:1n-9-nervonic acid), and PUFA—polyunsaturated fatty acids: C16:2n-4, C16:3n-4; C16:4n-1; C18:2n-6-*trans* –linoleic acid; C18:2n-6—linoleic acid; C18:3n-3-ɑ-linolenic acid; C18:3n-4; C18:3n-6-*gamma* linolenic acid; C18:4n-1; C18:4n-3—stearidonic acid; C20:2n-6—eicosadenoic acid; C20:3n-3—eicosatrienoic acid; C20:3n-6—*dihomo*-γ-linolenic acid; C20:4n-3—eicosapentenoic acid; C204n-6—arachidonic acid; C20:5n3—eicosaptenoic acid, C22:4n-6—adrenic acid; C22:5n-3—docosapentanoic acid; and C22:6n-3—docosahexanoic acid). The total SFA, MUFA, and PUFA were calculated based on the sum of the GC-determined afferent fatty acids.

### 2.5. Qualitative, Nutritional, Metabolic and Energy-Related Content Indexes Equations

The PUFA/SFA; n-6/n-3; LA/ALA; EPA + DHA; UI; NVI; IA; IT; HH; HPI, FLQ, EI, TI, ∆-9-desaturase(18:0); ∆-9-desaturase (16:0 + 18:0); and ∆-5-desaturase + ∆-6-desaturase, AI, INQ, Quanti n-3, HFI 1 and 2 were calculated according to formulas proposed recently by Dal Bosco et al. 2022 [14], as presented here: https://doi.org/10.3390/nu14153110 “URL (accessed on 9 November 2022)”. 

### 2.6. Experimental Design

This study aimed to evaluate the nutritional quality of sheep meat from animals traditionally raised in the NE area of Romania by providing information on the intrinsic quality of the lipid content in essential fatty acids. The nutritional evaluation aimed to establish the dry matter content and organic substances such as proteins and lipids, mineral substances, and energy value, serving to evaluate the possible differences in composition between the muscle regions of the studied sheep populations as a result of the imprinted variations in age and muscle region. This study also aimed to update the lipid nutritional indexing in sheep meat by exploring the nutritional properties by comparing the *Karakul* breed at different growth stages (lambs vs. adult sheep) and for each growth stage, the influence of the studied muscle regions (*Longissimus dorsi* × *Triceps brachii*) was analyzed. The essential fatty acid profile allows the identification of the rational use of lipid optimization in healthy human nutrition. Thus, it was intended to establish valid considerations about the nutritional effects of meat from different anatomical regions and from different age categories on human health to provide a useful assessment of *Karakul* genotypes and good practices.

### 2.7. Statistical Analysis

The data were processed using Microsoft Excel and the main statistical descriptors (mean values, variance, standard error of mean) and variance analysis using ANOVA, Single Factor, and Fisher tests were performed. The test consists of a comparison between calculated values and table values for Fα, where α takes values of 0.05, 0.01, and 0.001. If the value is lower than the table value for the 0.05 step, the differences between the values are not significant (n.s.). If the value is higher than the table value for the 0.05 step but less than the table value for the 0.01 step, the differences between the values are significant (*). If the value is higher than the table value for the 0.01 step but is less than the table value for the 0.001 steps, the differences between values are distinctly significant (**). If the value is higher than the table value for the 0.001 steps, then the differences between the values are highly significant (***).

## 3. Results and Discussion

### 3.1. Chemical Composition of Sheep Meat

Meat is the body’s main energy–plastic material and is one of the most representative sources of nitrogenous material with biological value. Lipids and carbohydrates are the main sources of energy, and proteins are the main source of plastids. As meat is considered a biological product, intense biochemical processes take place from collection to individual consumption [29].

Nutritional value is reflected in the chemical composition, particularly in the proportion and quality of the constituent chemical elements that play a determining role in human nutrition (proteins, lipids, and minerals). In terms of meat quality, the main factor is the muscle itself and the interdependence that exists at a macromolecular and micromolecular level between the biochemical and physicochemical constituents of one or more muscles. Variations in chemical composition can be attributed to the combined effects of pre-slaughter factors such as gender, breed, age, and diet and also post-mortem factors such as chilling and storage conditions.

Muscle samples from lambs revealed lower values for TDM (26.15%) in *Longissimus dorsi* and *Triceps brachii* (26.28%) compared to increased values (*p* ˃ 0.001) in adult sheep samples (Table 1). Similarly, the values noted for proteins, with the highest content (21.69% in *Longissimus dorsi*). Conversely, the lipid content analyses highlighted increased values (*p* ˃ 0.001) for adult sheep samples, (7.22 in *Longissimus dorsi* and 6.3% for *Triceps brachii*) compared to those found in lambs (*p* ˃ 0.001) (2.77% in *Longissimus dorsi* and 3.4 in *Triceps brachii*) (Table 1).

### 3.2. Fatty Acid Composition in Sheep Meat

Fats are the main compact energy source in the body and provide some important nutrients such as essential fatty acids, which act as structural elements in cell membranes, give palatability and flavour to meat, and in appropriate proportions, are the essential components of any balanced diet. Therefore, from a compositional point of view, a pragmatic analysis of these constituents is necessary to assess nutritional benefits.

Muscle samples from sheep carcasses presented lipids whose composition in saturated fatty acids showed increased values for C18:0 and C16:0 in all muscle groups that were analyzed. The mean values for C18:0 defined a lower limited range of 3.6 mg/g SU (*Triceps brachii* muscle/lambs). For C16:0, the limits of mean values materialized a difference of 14.4 mg/g SU (3.69 in *Triceps brachii* muscle, lambs vs. 18.1 mg/g SU in *Triceps brachii* muscles from adult sheep) (Table 2). Overall, with respect to saturated fatty acids, the specific composition of each muscle revealed that in the *Longissimus dorsi* muscle, the upper extremes of the means corresponding to each fatty acid were recorded in samples collected from adult sheep carcasses, except C12:0, C14:0, C15:0, C20:0, C21:0, and C22:0. In the *Triceps brachii* muscle, the upper extremes of the means corresponding to each fatty acid were recorded in most of the samples collected from carcasses of adult sheep except for C8:0 (Table 2).

For total MUFA, the highest amount was held by C18:1n-9 *cis*, whose average values defined a range delimited by 11.06 mg/g TDM (*Longissimus dorsi* m. from adults) and 28.1 mg/g TDM (*Triceps brachii*, m. from adults), with a difference about 17.04 mg/g TDM. Quantitatively, it was followed by C18:1n-9 *trans*, whose mean values defined a range of 1.38 mg/g TDM (*Longissimus dorsi* m., in adults) and 3.22 mg/g TDM (*Triceps brachii* m., in adults) followed by C15:1 and C16:1n-7. Within the MUFA, *Longissimus dorsi* from adults showed higher values for C14:1, C17:1, and C18:1n-9 *trans*, C18:1n-9 *cis*, and C22:1n-9 vs. lamb values. This trend was also maintained for *Triceps brachii*, except for C22:1n-9 where higher mean values were recorded in lambs, adding the parameters C16:1n-7 and C18:1n-7 with higher mean values in adults (Table 2).

Comparing the results obtained for MUFA, it was observed that they fall within the range of 36.6–40.4 mg/g, as obtained in other research in the literature [24,30].

The content of PUFA in the sheep meat was described by a wide range of variation in levels, the polyunsaturated lipid fractions with higher values being represented by C18:2n-6, whose mean values defined a lower bounded range of variation of 3.87 mg/g TDM (*Longissimus dorsi* m., from adults) and upper of 8.58 mg/g TDM (*Triceps brachii* m., from adults) followed by C18:3n-3. The rest of the PUFA recorded subunit values except for C20:04n-6 and C20:5n-3 in *Triceps brachii* from adults (3.1 mg/g TDM and 1.21 mg/g TDM). Both *Longissimus dorsi* and *Triceps brachii* collected from adult sheep showed higher mean values for major fatty acids.

Most SFA, MUFA, and PUFA can be synthesized by the body, except n-3 and n-6, which are considered essential for the proper function of the body. Their supply to the human body is provided through the daily food ration. The two types of PUFA incorporated in muscle tissues play a favourable role in preventing or reducing human diseases [4].

It is observed that n-6PUFA recorded a higher content than n-3PUFA, which can adjust inflammation by competing with n-6 metabolites for the incorporation of phospholipids into the membrane of immune system cells. *Triceps brachii* from lambs revealed lower values of n-6 and n-3PUFA compared to adult sheep, except for the n-3PUFA values recorded in *Longissimus dorsi*, which were higher in lamb (8.07% in lamb vs. 7.07% in adult sheep).

Research results indicate that slaughtering sheep at different ages determines variable values of SFA, MUFA, PUFA, and the n-6/n-3 ratio in *Longissimus dorsi* and *Triceps brachii* muscles. Thus, it was observed that with advancing age for *Longissimus dorsi*, the SFA content remains relatively constant (39.66% in lambs and 39.5% in adults), with decreases in PUFA content (25.14% in lambs vs. 24.18% in adults) (Table 2) and increases in MUFA content (35.18% in lambs vs. 36.3% in adults), while *Triceps brachii* m. showed a decrease in SFA percentage (45.57% in lambs vs. 41.95% in adults), an increase in PUFA content (17.8% in lambs vs. 22.57% in adults) and a decrease in MUFA percentage (36.59% in lambs and 35.46% in adults).

During the development of intramuscular fat deposits, it was observed that SFA and MUFA showed a more pronounced amplification compared to PUFA, highlighting repercussions for the decline in PUFA content and, therefore, the PUFA/SFA ratio. These differences are influenced by the genetic and dietary factors of the animals, which can be observed by the large range of variation in the values obtained for the fatty acid analysis of the intramuscular lipid constitution. The SFA content of total fatty acids in the samples collected from sheep carcasses had a lower limit of 39.5% TFA (*Longissimus dorsi*, adult sheep) and a higher limit of 45.57% TFA (*Triceps brachii* m., lamb meat). The MUFA content of total lipids in sheep meat ranged from 35.18% (*Longissimus dorsi*, lamb) to 36.59% (*Triceps brachii*, lambs). Lipid quality was assessed primarily by the percentage of PUFA (TFA), which ranged from 17.82% (*Triceps brachii* m.) to 25.14% (*Longissimus dorsi* m.), both extremes corresponding to samples from the lambs (Table 2). The PUFA/SFA ratio, as well as that of n-6/n-3, are two important parameters in the evaluation of meat lipids, the sheep meat being characterized by lipids whose PUFA/SFA ratio varied between 0.4 to 0.68 and the n-6/n-3 ratio showed values in the range 1.91–3.43% (Table 2). The results of this research describe meat with a higher mean PUFA content compared to those from sheep fed a PUFA-rich diet. Despite the perception that the lipid profile of the animals is largely made up of SFA, approximately 60% of TFA in the studied sheep meat is unsaturated (Table 2). Compared to data presented in the literature on the fatty acid content of sheep meat, the results of this research indicated that the percentage of SFA for *Triceps brachii* m. (41.96–45.57%) falls within the range (40.54–46.49%) [24,30]; in contrast, those for *Longissimus dorsi* showed a slightly lower range of values (39.5–39.67%), which is explained by the provided diet, which was a green meal. Thus, a decrease in the percentage of SFA and an increase in the PUFA percentage is observed, due to the abundant presence of linoleic acid (C18:3n-3) in the green mass, thus the sheep storing this acid in significant quantities in their tissues.

Regarding the content of sheep meat in essential fatty acids, it was observed that values of PUFA are in the range of 10.81–14.97%, with values showing significant variation within the range. The results obtained for n-3PUFA contained in *Longissimus dorsi* m. (from both categories of sheep taken in the study) show superiority (7.07–8.07%) to the quoted values (4.25–6.24%), and those contained in *Triceps brachii* m. from carcasses of adult sheep (5.77%) fall within the quoted range, except for the values obtained for the *Triceps brachii* collected from the lamb carcasses which were slightly lower (3.34%). Sheep meat has a high percentage of n-3PUFA, thus the consumption of this product is recommended as it has a positive impact on cardiovascular disease [14]. The important fatty acids with health benefits such as vaccenic acid (18:1n-7) [31] and long-chain PUFA n-3 appear in the Karakul breed. Other breeds studied by [32] do not have evidence of the presence of vaccenic acid.

The PUFA/SFA ratio shows values within the range of 0.12–0.54 specified in the literature [33,34,35,36,37]. Considering the recommended PUFA/SFA ratio is 0.45 [33], it can be argued that sheep offer meat that covers the requirements of a complete diet. The values obtained for the n-6/n-3 ratio in the range (1.91–2.5) are in accord with those obtained by researchers (1.03–2.49) such as [34,35,36,37]. The exceptions are those obtained for *Triceps brachii* m. collected from lamb carcasses, with a 3.43 ratio value, which through consumption, may be associated with an increased risk towards atherosclerosis and coronary heart disease.

The recommended value for the n-6/n-3 ratio is 2 [38]. n-6/n-3 ratio values of 1.91–2.55 were recorded for *Longissimus dorsi* m. (lambs and adult sheep), which is in agreement with those found by [34,39] who analysed meat from sheep fed with a green meal. This advantage of sheep meat from animals fed with a green meal is very important as it compensates for the low values of the PUFA to SFA ratio. Meat from ruminants and fatty fish are the only significant dietary sources of PUFA that have 20 and 22 carbon atoms in their structure [34,39].

### 3.3. Qualitative, Nutritional, Metabolic, and Energy Indexes

Based on the fatty acid values, four series of indexes were calculated including qualitative, nutritional, metabolic, and energy for the two muscular regions (*Longissimus dorsi* and *Triceps brachii*) from lambs and adult sheep.

The balance of n-6 and n-3 essential fatty acids is important in the prevention and treatment of coronary artery disease, hypertension, diabetes, arthritis, osteoporosis, autoimmune disorders, cancer, and mental health, together with the mechanisms involved [40]. Furthermore, food with a lower n-6/n-3 ratio is more desirable for reducing the risk of diseases mentioned before [14]. n-6 fatty acids are represented by linoleic acid (18:2n-6, LA) and n-3 fatty acids by ɑ-linolenic acid (18:3n-3, ALA). Linoleic acid can be converted into arachidonic acid and ɑ-linolenic acid in EPA and DHA, and existing competition between n-6 and n-3 fatty acids for enzyme desaturation—n-3 to n-6 for ∆-4 and ∆-6 desaturation is preferred [41].

The n-6/n-3 index shows that *Triceps brachii* meat from lambs and adult sheep (3.25 for lambs and 2.89 for adult sheep) has an equilibrated ratio of PUFA, whereas *Longissimus dorsi* has a much lower ratio (1.78 lambs and 1.95 for adult sheep) (Table 3).

Following this line of evolution (age at slaughter and muscle region in sheep) and analyzing the linoleic and ɑ-linolenic acid index, it can be observed how they are influenced. LA/ALA highlight that *Triceps brachii* meat from lambs is more interesting in a strictly qualitative context. Linoleic acid and ɑ-linolenic acid are essential PUFA with different effects on human health, which cannot be synthesized in the human body. Therefore, this index represents the first step in the formation of long-chain PUFA because they compete for the same metabolic pathway in desaturase and elongase reactions, which permits the synthesis of LC-PUFA [14]. The two indispensable precursors of PUFA, linoleic acid (LA—18:2n-6) and ɑ-linolenic acid (ALA—18:3n-3) have functional properties which are particularly linked to long-chain PUFA derivates found in all cellular membranes (structural role), and the production of oxygenated molecules resulting from their metabolism, linear or cyclic molecules, exert multiple roles as bioactive lipid mediators (eicosanoids, hydroxylated fatty acids, and docosanoids).

The PUFA bioconversion pathway is essential when the diet is chronically unbalanced. For example, it can take place in cases of too low linolenic acid intake and too high linoleic acid intake, limiting the synthesis of DHA (22:6n-3). An increase in arachidonic acid (AA, 20:4n-6) took place from the effects of metabolic competition that can produce a situation of subdeficiency resulting in the reduction in tissue concentrations of DHA and the abnormal appearance of a fatty acid substitution from the n-6 series, docosapentaenoic acid (22:5n-6). Linoleic, arachidonic, and docosahexanoic acids are fundamental constituents of membrane phospholipids. Together with cholesterol, they modulate the activity of a large number of intrinsic enzymes, transporters, receptors, and ion channels involved in inter and intracellular signalling within cell membranes [42]. This effect may facilitate the conformational changes necessary for the activity of intrinsic proteins [43]. As a nutritional recommendation for consumers, it is important to have a definite knowledge of the minimum requirements of linoleic and linolenic acid, as well as the main long-chain PUFA, in the main foods.

Several studies highlight the cardioprotective effects of EPA and DHA fatty acids, and long-chain n-3PUFA, which lower the levels of plasma triglycerides and reduce levels of both proinflammatory cytokines and chemokines [41]. The EPA and DHA sum gives higher values to *Triceps brachii* meat from adult sheep highlighting very significant differences (*p* > 0.001) compared to muscle regions in the lambs (Table 3). Both analyzed muscle region samples from adult sheep (*Longissimus dorsi* and *Triceps brachii*) are rich in PUFA. *Triceps brachii* muscle from adult sheep can be consumed to assure human health, with a positive impact on the reduction in the risk of cardiovascular disease, limiting the thrombosis process, inflammation, and hypertension and improving reproductive functions [13]. In addition to nutrition, a sedentary lifestyle and exposure to noxious substances interact with genetically controlled biochemical processes leading to chronic diseases [40]. *Triceps brachii* meat represents a rich source of essential fatty acids, such as EPA, DPA, and DHA that serve important cellular functions. They are a necessary component of human nutrition because the body does not have the biochemical pathway to produce these molecules on its own, particularly LA and ALA, which are introduced through the diet.

The UI index indicates the degree of unsaturation of FA, providing useful information regarding the shelf life of meat [14]. It shows the importance of the establishment of oxidative stability in human food, defining some oxidative protection strategies. Oxidative stress can be associated with the formation of lipid peroxides, processes that contribute to the ageing process and some diseases, such as atherosclerosis [44,45]. In the current research, *Longissimus dorsi* meat from adult sheep showed a lower value (*p* > 0.001) of UI concerning *Triceps brachii* from adult sheep, indicating a lower risk of fatty acid autoxidation. To assess the NVI and consumer health, the nutritional, atherogenicity, and thrombogenicity indexes, as well as hypocholesterolemic/hypercholesterolemic, health-promoting index, and flesh lipid quality were determined. Previous studies reported that total essential fatty acids and total desirable fatty acids (18:00 + MUFA + PUFA) have an important role in the biological activity of meat [46].

Both indexes of atherogenicity and thrombogenicity are desired to present smaller values than 1.0 and 0.5 [47] to assure a protective potential for coronary artery health. In this study, it was observed that meat from lambs presented higher values for IA than those from adult sheep. It has also been highlighted that the *Longissimus dorsi* and *Triceps brachii* from lambs and adult sheep demonstrated IA values lower than recommended values. These aspects are desirable for human health. The values obtained for IT varied between 0.78 (for *Longissimus dorsi*—from lambs) and 1.22 (for *Triceps brachii*—from lambs). However, IT values are close to the expected values. The thrombogenicity index highlights the tendency to form blood clots in vessels. Both indexes indicate the potential for stimulating platelet aggregation [15]. All obtained values for IT were slightly higher than the recommended value of 0.5. This good result points out the relationship between SFA and MUFA, considering the previous as proatherogenic (favouring adhesion of lipids to cells of the immune and circulatory system) and the pioneer of the activation of immunological cells, and so they adhere to the vessel wall, whereas the others are antiatherogenic, and finally, the levels of esterified fatty acids, cholesterol, and phospholipids are diminishing, thus preventing the occurrence of micro and macro-coronary diseases [3]. Thus, the smaller values of IA and IT present a greater protective potential for coronary artery disease. For lambs, no significant differences (*p* < 0.05) were observed through the two regional muscles analyzed. On the other hand, very significant differences were evidenced in IA values in each muscle influenced by age at slaughter. The IT of the *Longissimus dorsi* was not influenced by age at slaughter, evidenced by the lowest values for the thrombogenicity index (0.78 and 0.79)—this being acceptable from a human health point of view. *Triceps brachii* from lambs present high values for IT, at 1.22. The muscle studied in this research showed an IA and IT of 0.44–0.67 and 0.78–1.22, respectively. These results are in close agreement with those calculated based on the fatty acids in other studies for other species: for goose (IA = 0.36 and IT = 0.7), [15] for rabbit meat (IA = 0.9 and IT = 1.19) [48], for chicken (IA = 0.49 and IT = 1.14) [41], for turkey (IA = 0.47–0.78 and IT = 0.62–0.91) [24,42], for beef (IA = 0.6 and IT = 1.86) [49], and pork (IA = 0.47 and IT = 1.12) [50]. Compared to other kinds of lamb breeds (IA = 0.9 and IT = 0.87) [6], the values for the lambs in this study are much lower.

Hypocholesterolemic/hypercholesterolemic (HH) highlights the relationship between the fatty acid content of the meat and the plasma low-density lipoproteins relating to the hypocholesterolemic fatty acids (18:1 n-9 and PUFA) and hypercholesterolemic fatty acids (12:0, 14:0, 16:0). The HH index can serve to assess the cholesterolemic effect of lipids. In this study, the values of HH range between 2.17 and 2.67. Nutritionally higher HH values are considered beneficial for human health [3]. Since lipid deposits differ among breeds, this index seems to be mainly affected by the age at slaughter and muscle regions, highlighting very significant differences *p* ˃ 0.001. Different values for the HH index indicate the variant effects on cholesterol metabolism: higher values are considered more beneficial for human health. Compared to those found in other studies (1.55–1.57) [6], it can be observed that the values from this study are the highest but close to those found by [3] for goose (2.6–2.82). In other studies [44], comparing the meat from four breeds of suckling lambs revealed values for H/H of between 1.88 and 2.36, close to those obtained in this study. Higher values for the HH index were obtained for duck meat (3.5 [51]), marine fish fillets (3.1 [52]), and crab fillets (3.4 [53]). The HH values obtained for lambs and adult sheep in this study were better regarding the risk of atherosclerosis in comparison to rabbit (1.2 [48]), chicken (1.8 [54]), bovine (1.8 [49]), and swine (2.4 [55]).

The age at slaughter revealed very significant differences (*p* ˃ 0.001) for the ∆5-/∆6–Desaturase complex for *Triceps brachii* muscles, with superior values for the samples collected from adult sheep. Statistical analysis revealed significant differences (*p* ˃ 0.001) according to the muscle region with higher values for *Triceps brachii*. Thus, it is observed that adult sheep synthesize larger amounts of LC-PUFA from precursors, arguably indicated by the most valid tool for verifying the capacity to synthesize LC-PUFA, ∆5-/∆6–Desaturase. *Triceps brachii* represent a muscle region with a double efficiency in LC-PUFA synthesis, especially in adult sheep (Table 3).

The health-promoting index (HPI) allows the identification of the effects of fatty acids on cardiovascular diseases, representing the inverse of the index of atherogenicity, thus highlighting inverse values compared to IA (1.5 to 2.27, respectively, in *Triceps brachii*—adult sheep and *Longissimus dorsi*—adult sheep) (Table 3). FLQ allows the evaluation of the quality of EPA + DHA, calculated as the percentage of TFA compared to saturated fatty acids. The *Triceps brachii* of adult sheep showed an almost five times higher value than the same muscle from lambs. *Longissimus dorsi* muscle does not show a difference (*p* ˂ 0.05) regarding age at slaughter. The muscle region highlighted very significant differences (*p* ˃ 0.001) in both age categories at slaughter.

Based on critical reports from the scientific literature, other authors [14] elaborated on two indexes (healthy fatty index 1 and 2). The essence of those indexes was to give different weights to the various types of fatty acids, reporting on their healthy properties. HFI 1 expressed all fatty acids with less impact on human health, reported as the numerator and multiplied with empirical constants elaborated on by Ulbricht and Southgate [56], 8 for n-3, 4 for n-6, and 2 for MUFA, to consider the nutritional and health impact on various classes reported in total lipids. In this study, *Longissimus dorsi* from lambs and *Triceps brachii* from adult sheep showed the highest values for HFI 1 (20.99 and 24.74, respectively). Both muscle regions and age at slaughter showed very significant differences (*p* ˃ 0.001) in HF 1 values (Table 3). From the analysis of the HFI 2, there is no doubt that *Longissimus dorsi* was not influenced by age at slaughter (*p* ˂ 0.05). Analyzing lamb meat, it can be observed that *Longissimus dorsi* presents higher values for HFI 2 (*p* ˃ 0.001) (2.76 vs. 1.7 on *Triceps brachii* muscle). The same aspects can be observed for adult sheep with higher values for *Longissimus dorsi* (*p* ˂ 0.05) (Table 3). HFI have higher values in very lean meat [14]. Flesh lipid quality, the health-promoting index, and the healthy fatty index make extremely rare appearances when sheep meat is discussed.

Palmitic and oleic acids represent the principal total fatty acids, the ratio of (C18:0 + C18:1)/C16:0 being an important criterion in the prediction of meat lipid quality defined as the nutritional value of meat [32]. Other authors [32,57] obtained slightly lower values compared to the results of this study, which ranged between 2.4 and 2.56. These same authors reported that the NVI found values in the literature ranging between 2 and 3 in lamb meat. The values found in this study are lower than those reported by [58] for *Turkish* lambs (2.49–2.67). The highest value of NVI was evidenced in the results of this study for the *Longissimus dorsi* muscle from adult sheep and *Triceps brachii* from lambs (Table 3). This was caused by the highest proportions of 18:0 and 18:1n-9, and the lowest values of 16:0.

The results of this study highlight how complex it can be to index fatty acids to estimate the nutritional properties and health benefits of sheep meat.

## 4. Conclusions

The *Karakul* breed has a significant role in sheep meat production, presenting high nutritional value. The research results indicate that qualitatively, *Longissimus dorsi* are superior to *Triceps brachii* due to a lower content in SFA (39.66% in *Longissimus dorsi* vs. 45.57% in *Triceps brachii*).

The n-6/n-3 index shows that *Triceps brachii* meat from lambs and adult sheep has an equilibrated PUFA ratio, whereas *Longissimus dorsi* has a lower ratio. Therefore, reporting antagonistic effects of n-3, n-6, and LC-PUFA in their proportional ratios with each other, which must be close to 4/1, *Triceps brachii* from lambs could play a significant role in regulating body homeostasis of inflammation and anti-inflammation, vasodilation and vasoconstriction, and platelet aggregation and anti-aggregation.

*Triceps brachii* meat represents a rich source of essential fatty acids, such as EPA, DPA, and DHA that serve important cellular functions. The data on sheep meat quality and sanogenic lipid indicators are important, offering a positive impact on developing marketing strategies that would allow both individuals/groups of farmers, processors, and local authorities to promote the *Karakul* breed based on objective analyses.

## Figures and Tables

**Table 1:** Chemical quality indicators (%) of *Longissimus dorsi* and *Triceps brachii* muscles from different age categories of sheep (*Karakul* breed).

Specification	*Ld*		ANOVA L × AS	*Tb*		ANOVA L × AS
	L	AS		L	AS	
H_2_O	73.85 ± 0.07	70.05 ± 0.36	***	73.72 ± 0.62	71.08 ± 0.12	***
TDM	26.15 ± 0.07	29.95 ± 0.36	***	26.28 ± 0.62	28.92 ± 0.12	***
Proteins	21.69 ± 0.05	20.95 ± 0.09	***	21.18 ± 0.11	20.81 ± 0.09	*
Lipids	2.77 ± 0.04	7.22 ± 0.29	***	3.40 ± 0.58	6.3 ± 0.25	***
TMS	1.1 ± 0.02	1.09 ± 0.01	n.s.	1.18 ± 0.02	1.14 ± 0.01	n.s.
NFE	0.59 ± 0.03	0.69 ± 0.05	n.s.	0.52 ± 0.04	0.67 ± 0.02	**
GE	152.5 ± 0.5	190.9 ± 3.1	***	155.05 ± 5.7	181.3 ± 1.7	***

* H_2_O—total water; TDM—total dry matter; TMS—total mineral substances; NFE—nonnitrogenous extractive substances; GE—energetic value (kcal/100 g); L—lambs; AS—adult sheep; Ld—*Longissimus dorsi* muscle; Tb—*Triceps brachii* muscle; n.s.—no significant difference (*p* ˂ 0.05); *—significant difference (*p* ˂ 0.01); **—distinct difference (*p* ˂ 0.001); ***—very significant difference (*p* ˃ 0.001).

**Table 2:** Fatty acid contents (mg/g TDM) of the *Longissimus dorsi* and *Triceps brachii* muscles from different age groups.

Fatty Acids	*Ld*		ANOVA L × AS	*Tb*		ANOVA L × AS	L	AS
	L	AS		L	AS		Ld × Tb	Ld × Tb
C8:0	0.01 ± 0.001	0.0179 ± 0.003	**	0.02 ± 0.002	0.00	***	***	***
C10:0	0.05 ± 0.004	0.050 ± 0.006	n.s.	0.028 ± 0.006	0.211 ± 0.03	***	**	***
C12:0	0.099 ± 0.01	0.042 ± 0.01	***	0.041 ± 0.009	0.584 ± 0.1	***	***	***
C14:0	1.05 ± 0.11	0.667 ± 0.08	**	0.474 ± 0.085	4.441 ± 0.41	***	***	***
C15:0	0.14 ± 0.01	0.096 ± 0.01	*	0.07 ± 0.009	0.620 ± 0.067	***	***	***
C16:0	6.042 ± 0.4	7.29 ± 0.5	n.s.	3.695 ± 0.4	18.10 ± 0.8	***	***	***
C17:0	0.199 ± 0.01	0.74 ± 0.01	***	0.14 ± 0.02	1.412 ± 0.05	***	*	***
C18:0	4.748 ± 0.38	6.188 ± 0.4	*	3.632 ± 0.35	13.33 ± 0.74	***	*	***
C20:0	0.034 ± 0.002	0.027 ± 0.002	n.s.	0.023 ± 0.003	0.216 ± 0.003	***	*	***
C21:0	0.053 ± 0.001	0.046 ± 0.001	***	0.01± 0.002	0.17 ± 0.01	***	***	***
C22:0	0.053 ± 0.001	0.00	***	0.04 ± 0.002	0.204 ± 0.03	***	***	***
C23:0	0.014 ± 0.001	0.017 ± 0.001	n.s.	0.037 ± 0.002	0.115 ± 0.028	***	***	***
C24:0	0.023 ± 0.001	0.037 ± 0.001	***	0.04 ± 0.002	0.123 ± 0.01	***	***	***
**Total SFA, mg/g**	12.51 ± 0.93	15.21 ± 0.08		8.25 ± 0.06	39.52 ± 0.18			
**TotalSFA, (% TFA)**	39.66 ± 1.1	39.5 ± 0.78		45.58 ± 0.85	41.95 ± 1.05			
C14:1	0.038 ± 0.003	0.039 ± 0.01	n.s.	0.027 ± 0.003	0.432 ± 0.07	***	*	***
C15:1	0.488 ± 0.02	0.441 ± 0.02	n.s.	0.435 ± 0.03	0.00	***	n.s.	***
C16:1n7	0.387 ± 0.041	0.381 ± 0.03	n.s.	0.221 ± 0.03	0.836 ± 0.08	***	***	***
C17:1	0.147 ± 0.001	0.372 ± 0.01	***	0.039 ± 0.002	0.488 ± 0.02	***	***	***
C18:1n9t	0.488 ± 0.02	1.383 ± 0.026	***	0.119 ± 0.002	3.217 ± 0.207	***	***	***
C18:1n9cis	9.257 ± 0.8	11.06 ± 0.73	n.s.	5.69 ± 0.57	28.1 ± 1.28	***	***	***
C18:1n7	0.108 ± 0.01	0.102 ± 0.02	n.s.	0.101 ± 0.01	0.23 ± 0.06	***	n.s.	***
C22:1n9	0.02 ± 0.002	0.03 ± 0.002	**	0.018 ± 0.001	0	***	n.s.	***
C24:1n9	0.0018 ± 0.002	0.034 ± 0.002	***	0.031 ± 0.003	0.104 ± 0.01	***	***	***
**Total MUFA, mg/g**	10.935± 0.09	13.84 ± 0.09		6.68 ± 0.07	33.4 ± 0.24			
**MUFA, (% TFA)**	35.18 ± 0.93	36.31 ± 0.58		35.59 ± 0.58	35.46 ± 1.47			
C16:2n4	0.312 ± 0.02	0.443 ± 0.03	**	0.195 ± 0.02	0.808 ± 0.05	***	**	***
C16:3n4	0.173 ± 0.01	0.19 ± 0.01	n.s.	0.106 ± 0.01	0.652 ± 0.1	***	**	***
C16:4n1	0.28 ± 0.01	0.297 ± 0.07	n.s.	0.313 ± 0.02	0.965 ± 0.1	***	n.s.	***
C18:2n6t	0.124 ± 0.001	0.276 ± 0.02	***	0.06 ± 0.01	0.282 ± 0.03	***	***	n.s.
C18:2n4	0.072 ± 0.01	0.09 ± 0.01	n.s.	0.055 ± 0.01	0.171 ± 0.04	***	n.s.	**
C18:2n6	3.269 ± 0.34	3.87 ± 0.2	n.s.	1.223 ± 0.08	8.582 ± 0.89	***	***	***
C18:3n3	1.525 ± 0.2	1.577 ± 0.2	n.s.	0.254 ± 0.02	2.41 ± 0.2	***	***	***
C18:3n4	0.03 ± 0.002	0.04 ± 0.004	n.s.	0.015 ± 0.003	0.073 ± 0.01	***	***	**
C18:3n6	0.13 ± 0.02	0.161 ± 0.001	n.s.	0.06 ± 0.003	0.093 ± 0.003	***	***	**
C18:4n3	0.26 ± 0.03	0.33 ± 0.03	n.s.	0.17 ± 0.02	0.288 ± 0.02	***	*	n.s.
C20:2	0.003 ± 0.0002	0.06 ± 0.002	***	0.001 ± 0.0004	0.092 ± 0.01	***	*	**
C20:2n6	0.07 ± 0.005	0.08 ± 0.004	n.s.	0.052 ± 0.005	0.073 ± 0.01	*	*	n.s.
C20:3n6	0.04 ± 0.002	0.053 ± 0.003	***	0.032 ± 0.003	0.67 ± 0.1	***	n.s.	***
C20:4n3	0.106 ± 0.01	0.152 ± 0.01	*	0.104 ± 0.01	0.284 ± 0.02	***	n.s.	***
C20:4n6	0.414 ± 0.01	0.452 ± 0.01	n.s.	0.36 ± 0.02	3.102 ± 0.6	***	*	***
C20:5n3	0.121 ± 0.01	0.17 ± 0.02	n.s.	0.001 ± 0.0002	1.214 ± 0.2	***	***	***
C22:4n6	0.152 ± 0.009	0.194 ± 0.05	n.s.	0.133 ± 0.01	0.304 ± 0.03	***	n.s.	n.s.
C22:5n3	0.353 ± 0.02	0.382 ± 0.03	n.s.	0.07 ± 0.02	0.341 ± 0.02	***	***	n.s.
C22:6n3	0.009 ± 0.003	0.03 ± 0.01	n.s.	0.03 ±0.02	0.92 ± 0.17	***	n.s.	***
**∑ PUFA**	7.44 ± 0.003	8.847 ± 0.03		3.234 ± 0.01	21.324 ± 0.14			
**∑ PUFA, (%TFA)**	25.14 ± 1.9	24.18 ± 1.19		17.82 ± 0.94	22.57 ± 2.25			
**n-3**	2.365 ± 0.05	2.61 ± 0.058		0.59 ± 0.01	4.537 ± 0.09			
**n-3, (%TFA)**	8.07 ± 0.8	7.07 ± 0.6		3.34 ± 0.3	5.77 ± 0.6			
**n-6**	4.199 ± 0.07	5.08 ± 0.057		1.92 ± 0.02	13,106 ± 0.33			
**n-6, (%TFA)**	14.28 ± 1.3	14.08 ± 0.8		10.81 ± 0.6	13.87 ± 1.6			
**n-6/n-3, mg/g**	1.775	1.95		3.20	2.88			
**n-6/n-3,** **(%TFA)**	1.91 ± 0.13	2.55 ± 0.38		3.43 ± 0.27	2.35 ± 0.13			
**PUFA/MUFA**	0.68	0.64		0.48	0.64			
**%TFA**	0.68 ± 0.07	0.63 ± 0.04		0.4 ± 0.02	0.54 ± 0.06			

L—lambs; AS—adult sheep; Ld—*Longissimus dorsi* muscle; Tb—*Triceps brachii* muscle; SFA—saturated fatty acids; MUFA—monounsaturated fatty acids; PUFA—polyunsaturated fatty acids; TFA—total fatty acids; n.s.—no significant difference (*p* ˂ 0.05); *—significant difference (*p* ˂ 0.01); **—distinct difference (*p* ˂ 0.001); ***—very significant difference (*p* ˃ 0.001).

**Table 3:** Qualitative, nutritional, metabolic, and energy indexes for *Longissimus dorsi* and *Triceps brachii* muscles from different age groups of sheep.

Indexes	*Ld*		*Triceps brachii*		*Ld*	*Tb*	L	AS
	L	AS	L	AS	L × AS	L × AS	Ld × Tb	Ld × Tb
**Qualitative**					**			
PUFA/SFA	0.68	0.64	0.48	0.64	***	***	***	n.s.
n-6/n-3	1.78	1.95	3.25	2.89	***	***	***	***
LA/ALA	2.14	2.45	4.81	3.56	**	***	***	***
EPA + DHA	0.13	0.2	0.03	2.13	***	***	***	***
UI	7.2	4.4	5.1	10.2		***	***	***
**Nutritional**					***			
NVI	2.4	2.56	2.56	2.47	***	***	***	***
IA	0.56	0.44	0.57	0.67	n.s.	***	n.s.	***
IT	0.78	0.79	1.22	0.96	***	***	***	***
HH	2.41	2.67	2.17	2.29	***	***	***	***
HPI	1.78	2.27	1.76	1.5	n.s.	***	n.s.	***
FLQ	3.86	3.82	1.22	6.29		***	***	***
**Metabolic Indexes**					**			
EI	78.58	84.88	98.29	73.65	***	***	***	***
TI	575.4	1092.9	779.5	407.5	n.s.	***	***	***
∆9-Des (C18:1)	67.24	66.79	61.53	70.14	n.s.	***	**	*
∆9-Des (C16, C18)	48.4	48.7	45.1	50.6	n.s.	**	*	n.s.
∆5- Des + ∆6–Des	0.23	0.23	0.42	0.54	***	***	***	**
AI	1.55	1.66	2.36	1.88		**	***	*
**Energy-related content indexes**					***			
EPA + DHA	0.13	0.2	0.03	2.13	n.s.	**	***	*
INQ	0.0003	0.0003	0.003	0.003	***	n.s.	***	***
n-3 Index	0.85	0.36	0.18	0.72	***	***	***	**
HFI1	20.99	9.62	7.69	24.74	n.s.	***	***	***
HFI2	2.76	2.7	1.7	2.48		***	***	n.s.

* PUFA/SFA—polyunsaturated fatty acids/saturated fatty acids; LA—linoleic acid; ALA—ɑ-linolenic acid; EPA—eicosapentaenoic acid (C20:5n-3); DHA—docosahexaenoic acid (C22:6n-3); UI—unsaturation index; NVI—nutrition value index; IA—index of atherogenicity; IT—index of thrombogenicity; HH—hypocholesterolemic/hypercholesterolemic ratio; HPI—health-promoting index; FLQ—flesh lipid quality; EI—elongase; TI—thioesterase; ∆9-Des (C18:1)—∆9-Desaturase (C18:1), ∆9-Des (C16, C18)-∆9—Desaturase (C16:1, C18:1); ∆5-Des + ∆6-Des—D5-desaturase+ D6-desaturase; AI—activity index; INQ—index of nutritional quality; HFI1—healthy fatty index1; HFI2—healthy fatty index2 n.s.—no significant difference (*p* ˂ 0.05); *—significant difference (*p* ˂ 0.01); **—distinct difference (*p* ˂ 0.001); ***—very significant difference (*p* ˃ 0.001).

## Data Availability

The data presented in this study are available within the article.
